# Targeting the CD146/Galectin-9 axis protects the integrity of the blood–brain barrier in experimental cerebral malaria

**DOI:** 10.1038/s41423-020-00582-8

**Published:** 2020-11-17

**Authors:** Hongxia Duan, Shuai Zhao, Jianquan Xiang, Chenhui Ju, Xuehui Chen, Irene Gramaglia, Xiyun Yan

**Affiliations:** 1 Key Laboratory of Protein and Peptide Pharmaceutical, Institute of Biophysics, Chinese Academy of Sciences, Beijing, 100101 China; 2 Laboratory of Biochemistry and Molecular Biology, School of Basic Medical Sciences, Southwest Medical University, Luzhou, 646000 Sichuan China; 3 La Jolla Infectious Disease Institute, San Diego, CA USA; 4 Joint Laboratory of Nanozymes in Zhengzhou University, School of Basic Medical Sciences, Zhengzhou University, Zhengzhou, 450001 Henan China

**Keywords:** CD146, BBB, experimental cerebral malaria, Infectious diseases, Mechanisms of disease

## Abstract

Cerebral malaria (CM) is a life-threatening diffuse encephalopathy caused by *Plasmodium falciparum*, in which the destruction of the blood–brain barrier (BBB) is the main cause of death. However, increasing evidence has shown that antimalarial drugs, the current treatment for CM, do little to protect against CM-induced BBB damage. Therefore, a means to alleviate BBB dysfunction would be a promising adjuvant therapy for CM. The adhesion molecule CD146 has been reported to be expressed in both endothelial cells and proinflammatory immune cells and mediates neuroinflammation. Here, we demonstrate that CD146 expressed on BBB endothelial cells but not immune cells is a novel therapeutic target in a mouse model of experimental cerebral malaria (eCM). Endothelial CD146 is upregulated during eCM development and facilitates the sequestration of infected red blood cells (RBCs) and/or proinflammatory lymphocytes in CNS blood vessels, thereby promoting the disruption of BBB integrity. Mechanistic studies showed that the interaction of CD146 and Galectin-9 contributes to the aggregation of infected RBCs and lymphocytes. Deletion of endothelial CD146 or treatment with the anti-CD146 antibody AA98 prevents severe signs of eCM, such as limb paralysis, brain vascular leakage, and death. In addition, AA98 combined with the antiparasitic drug artemether improved the cognition and memory of mice with eCM. Taken together, our findings suggest that endothelial CD146 is a novel and promising target in combination with antiparasitic drugs for future CM therapies.

## Introduction

Malaria is one of the most important mosquito-borne infectious diseases in humans worldwide that is caused by the parasite *Plasmodium falciparum*. Cerebral malaria (CM) is a deadly complication of malaria and is characterized by parasitemia, fever, and coma. The mortality rate of CM is as high as 30%, accounting for 90% of deaths from malaria. CM is especially dangerous in children. Moreover, 10–24% of surviving patients have long-lasting neurological sequelae and memory impairments.^1,2^ Clinical studies have demonstrated that although antimalarial drugs are effective at clearing *Plasmodium* parasites, they do little to protect against parasite-associated neuroinflammation and subsequent neurological sequelae. In addition, current adjunctive therapies targeting neuroinflammation or neurological sequelae, including hormone therapy,^3^ tumor necrosis factor alpha (TNFα) blockade,^4^ iron-chelating agent,^5,6^ anticonvulsant drugs,^7,8^ hypertonic solutions,^9^ and antioxidants,^10^ either have little effect on mortality or have considerable systemic side effects. Therefore, further elucidation of CM-meditated pathogenic features in central nervous system (CNS) inflammation may be helpful for developing immunotherapeutics to ameliorate CM-associated brain damage and mortality.^11^

Intense efforts have been made to understand the pathogenesis of CM. Increasing evidence from clinical samples and animal models have shown that disruption of the blood–brain barrier (BBB) is responsible for the development of CM.^12^ The disruption of the BBB in CM has been reported to lead to severe neurological complications, including intracerebral hemorrhage, electrolyte imbalance, and an increase in intracranial pressure, all of which ultimately result in CNS dysfunction and death. Therefore, measures to prevent or alleviate BBB dysfunction may provide an attractive additional treatment strategy. However, due to the limited understanding of the underlying molecular mechanisms of CM-induced BBB breakdown, most interventions in experimental models do not prevent lethal progression after the onset of experimental CM (eCM).

Previous studies have shown that the sequestration of parasitized red blood cells (pRBCs) by brain endothelial cells and excessive local inflammation caused by intracerebrally recruited leukocytes are associated with BBB dysfunction.^13,14^ The sequestration of pRBCs slows blood flow, leading to tissue perfusion injury and hypoxia. In addition, leukocytes such as CD8^+^ T cells are recruited, which release proinflammatory cytokines to promote the activation or apoptosis of BBB endothelial cells (BBBECs), resulting in BBB disruption, the subsequent infiltration of proinflammatory cells into the CNS, and ultimately neuropathological events, brain edema, or coma. Therefore, ideal agents for alleviating BBB dysfunction should specifically block pRBC sequestration and leukocyte recruitment while sparing the host’s protective immune response.

Cluster of differentiation 146 (CD146, also known as melanoma cell-adhesion molecule) is a member of the immunoglobulin superfamily and has been identified as a novel endothelial biomarker that acts as a vascular endothelial growth factor coreceptor and plays a key role in tumor-related angiogenesis.^15,16^ CD146 is primarily expressed at the intercellular junctions of endothelial cells. Recently, we reported that CD146 is a mediator of the interplay between endothelial cells and pericytes, promoting BBB development.^17^ During conditions of neuroinflammation, such as in multiple sclerosis (MS), CD146 is upregulated on BBBECs and promotes the transmigration of inflammatory cells into the CNS.^18^ In addition, CD146 is also expressed on some proinflammatory cells, such as T cells^19^ and macrophages,^20^ which are associated with the development of inflammation. Importantly, treatment with the anti-CD146 functional antibody AA98 ameliorates neuroinflammation but appears to not disturb the host’s protective immune response.^18,20^ Given the similarities in the pathophysiological features and BBB dysfunction in CM and MS, we sought to determine whether targeting CD146 alleviated neuroinflammation and BBB damage and improved survival in mice with late-stage eCM.

In the present study, we investigated the role of CD146 in the development of CM using various CD146 conditional-knockout mice and a murine eCM model that recapitulates human CM in many ways, including brain endothelial-cell activation, pRBC/leukocytic sequestration in the brain vasculature, and breakdown of the BBB. We found that CD146 expressed on BBBECs but not T cells or macrophages is a novel therapeutic target for eCM. Endothelial CD146 facilitated BBB dysfunction by promoting the sequestration of pRBCs in the brain vasculature and the recruitment of T cells to the CNS via interactions with galectin-9, a β-galactoside-binding lectin. Therapeutic treatment with the CD146 antibody AA98 blocked the development of eCM. As BBB breakdown is critical during CM development, our present findings provide new insights into additional therapeutic strategies for CM.

## Materials and methods

### Antibodies and reagents

The following antibodies and reagents were used in this study: anti-CD146 mAb AA98, AA98-PE, AA1-APC, AA1-PE (all generated in our laboratory^16,21,22^), anti-mouse CD31 (ab56299), anti-GAPDH (ab8245), recombinant Fc (Cat. no. 10702-HNAH), recombinant Gal-9-Fc (Cat. no. 11147-H01H), recombinant human TNFα (AF-300-01A), IFNγ (AF-300-02) and IL-1β (AF-200-01B), Cytokine & Chemokine 36-Plex Mouse ProcartaPlex Panel 1 A (Cat. no. EPX360-26092-901), PerCP/Cy5.5 anti-mouse CD3ε (Cat. no. 100327), Alexa Fluor 700 anti-mouse CD45.2 (Cat. no. 109822), APC/Cy7 anti-mouse Ly-6G/Ly-6C (Gr-1) (Cat. no. 108424), Brilliant Violet 510 anti-mouse CD4 (Cat. no. 100449), PE/Cy7 anti-mouse CD146 (Cat. no. 134714), anti-mouse F4/80 antigen eFluor 450 (Cat. no. 48-4801-82), anti-mouse CD11b APC (Cat. no. 17-0112-82), rat anti-mouse CD19 monoclonal antibody, PE (Cat. no. M10191-09D), PerCP/Cy5.5 anti-mouse Galectin-9 (Cat. no. 136111), MojoSort Mouse CD3 T cell isolation kit (Cat. no. 480023), RBC lysis buffer (10X) (Cat. no. 420301), LEAF purified anti-mouse IFN-γ (Cat. no. 505812), Giemsa stain solution (Cat. no. 32884-250 ML), Evans blue (Cat. no. E2129-10G), and artemether (Cat. no. 71963-77-4).

### Animals

All animal experiments were approved by the Institutional Biomedical Research Ethics Committee of the Institute of Biophysics at the Chinese Academy of Sciences (permit number: DWSWAQ(ABSL-2)201703). Animal experiments were performed in compliance with the guidelines for the care and use of laboratory animals. All mice were housed in a pathogen-free facility.

All conditional-knockout mice, including Tek^cre/+^CD146^floxed/floxed^ (CD146^EC-KO^) mice, Lck^cre/+^CD146^floxed/floxed^ (CD146^T-KO^) mice, and Lysm^cre/+^CD146^floxed/floxed^ (CD146^M-KO^) mice, were generated using a Cre/loxP recombination system. In brief, cre/+CD146^+/+^ mice (obtained from Jackson Laboratories) were crossed with CD146^floxed/floxed^ mice (obtained from the Nanjing Biomedical Research Institute of Nanjing University). The F1 cre/+CD146^floxed/+^ genotype was backcrossed with CD146^floxed/floxed^ mice to obtain cre/+CD146^floxed/floxed^ mice, which we refer to as CD146 conditional-knockout mice. CD146^floxed/floxed^ mice and cre/+ CD146^+/+^ (which we refer to as WT mice here) were used as controls in the eCM induction experiments. All genotypes were confirmed by PCR analysis.

Gal-9-knockout (Gal-9-KO) mice were generated using CRISPR-Cas9 gene-editing technology. Female C57BL/6 J mice ranging in age from 5–6 weeks were obtained from the Department of Laboratory Animal Science, Peking University Health Science Center.

### Experimental cerebral malaria model and treatment

The eCM model was established as previously described.^23^ In brief, a frozen aliquot of *P. berghei* ANKA strain-infected RBCs (pRBCs) (stored in the laboratory of Dr Henri C van der Heyde, La Jolla Bioengineering Institute) was intravenously (i.v.) injected into a resistant mouse (C57BL/6 mouse older than 12 weeks). One week later, parasitemia was assessed by Giemsa-stained thin blood films. Once the donor mouse reached a parasitemia level of ~15–25%, retro-orbital blood was collected in tubes containing citrate buffer (6.25 g of sodium citrate, 2 of H_2_O, 3.1 g of citric acid anhydrous, and 3.4 g of D-glucose in 250 ml of H_2_O). The blood was then diluted two times (1:10) in PBS (w/o Ca/Mg). The mice were weighed and tagged, and 10^6^ pRBCs were i.v. injected into each mouse in a volume of 0.2 ml.

Beginning on day 4 postinfection, the mice were weighed, and parasitemia was assessed every two days. Starting at day 6 postinfection, clinical scores were assessed by summing the gripping reflex and righting reflex on a scale of 0–5, in which 0 indicated no reflex and 5 indicated unimpaired, as previously described.^24^ The mice were also monitored daily.

For antibody treatment, 10 mg/kg AA98 (generated in our laboratory) or mIgG (Cat. no. I5381) was intraperitoneally (i.p.) injected at day 0, day 3 or day 5 after *P. berghei* ANKA infection. For combined treatment, artemether (30 mg/kg, dissolved in olive oil) was administered by the oral gavage every other day starting at day 4 for a total of five administrations, and AA98 (10 mg/kg) was injected i.p. every three days starting at day 4 for a total of three injections.

### Permeability assay

BBB permeability was measured using Evans blue (molecular weight 960.8, label albumin) as described previously.^17^ In brief, mice were i.v. injected with 200 μl of Evans blue solution (0.5% in PBS) at D6 after infection with PbA. Uninfected mice were used as controls. Three hours later, the mice were exsanguinated under anesthesia. The brains were removed and weighed. Formamide (500 μl) was added, and the samples were incubated for three days at 4 °C in the dark. After three days of formamide extraction, Evans blue absorption was measured at 620 nm (OD620).

### Mouse BBBEC isolation

Isolation of mouse BBBECs was performed as described previously.^18^ In brief, the collected mouse brain tissues were washed twice in 75% ethanol, followed by three washes with PBS. Finally, the large blood vessels of the meningeal tissue were removed with filter paper. The remaining tissues were chopped into pieces in DMEM and then incubated with 0.1% collagenase (w/v) at 37 °C for 1 h. The suspension was then centrifuged and resuspended in DMEM containing 25% BSA and centrifuged at 1,200 rpm for 40 min. The pellet was digested with 0.1% collagenase for 1 h. The suspension was harvested and then grown in EBM-2 media. For FACS analysis, the cultured cells were harvested and stained with antibodies against CD146 and CD31. The samples were then analyzed by flow cytometry.

### Isolation of CNS lymphocytes

Brain tissues were collected from anesthetized mice perfused with 40 ml of PBS. The collected brain tissues were ground in sterile PBS and then centrifuged. The pellets were resuspended in 30% Percoll cell-separation solution (Percoll stock solution was diluted with 10 × PBS at a ratio of 9:1 and was then diluted with 1 × PBS at a ratio of 7:3–30% to prepare the Percoll working solution). The suspensions were centrifuged at 2118 rpm for 15 min at room temperature. The supernatant and liquid were removed, and the pellets were collected and washed with PBS once the CNS leukocytes were obtained. The collected cells were used for flow cytometry.

### Luminex-technology multiplex analysis of serum proinflammatory factors

Fifty microliters of sera from healthy and eCM mice were collected on day 6 post-PbA injection, and proinflammatory factor profiles were characterized using the Cytokine & Chemokine 36-Plex Mouse ProcartaPlex Panel 1 A (Cat. no. EPX360-26092-901), according to the manufacturer’s instructions.

### Cell lines and plasmids

The mouse BBBEC cell line bEnd.3 and the mouse T cell line EL4 were used in this study and were purchased from ATCC. Knockdown of CD146 was performed using CD146 shRNA, which was constructed within the lentiviral vector pHS-ASR (SyngenTech). The target sequences were as follows: shRNA007: GCGGGAACCTGGTGAATATGA; shRNA008: GGATCACTACGTTGAGCTTCA; shRNA009: GCACAGCCATTGGTGGCAAAT; and NC-shRNA: GTAATTGTCAAATCAGAGTGCT. Overexpression of CD146 was performed using a pHS-AVC vector (SyngenTech). Knockdown of Gal-9 was performed using Gal-9 shRNA carried by the lentiviral vector LV3 (GenePharma). The target sequences were as follows: NC-shRNA: TTCTCCGAACGTGTCACGT and GAL-9-shRNA1: GATGGTGAACAAGAAATTCTT. Overexpression of Gal-9 was performed using an LV5 vector (GenePharma).

### RBC and T cell adhesion assay

Mouse bEnd.3 cells (1 × 10^5^) were seeded in 12-well plates. After the cells reached confluence, isolated RBCs or T cells (5 × 10^5^) from healthy mice or eCM mice (day 4 after PbA infection) were added. After 4 h of culture, the supernatant was discarded, and the cells were washed three times with PBS and collected for counting and flow cytometric analysis.

### Samples for IP tandem-mass spectrometry

After being washed twice with precooled PBS, equal amounts of stably transfected CD146-Flag bEnd.3 cells or control Flag-bEnd.3 cells and primary T cells isolated from healthy mice or eCM mice were mixed. The mixture was then lysed with CelLytic M-cell lysis buffer (C2978, Sigma) on ice for 30 min. After centrifugation at 13,000 rpm, the supernatant was placed in a new 1.5-ml tube, and 20 μl of EZview Red anti-FLAG M2 affinity gel (F2426, Sigma) was added and incubated on a mixer at 4 °C overnight. After centrifugation, the pellet was washed three times with TBST, and 200 μl of flag-peptide eluent was added and incubated at 4 °C for 2 h. After centrifugation at 3000 rpm for 2 min, the supernatant was collected, added to protein-sample loading buffer and boiled at 100 °C for 10 min. This sample was used for tandem-mass spectrometry analysis or Western blotting.

### Behavioral analysis

A Y maze and a Morris water maze were used to test the cognitive function of treated eCM mice.

The Y maze test was performed as previously described.^25^ The apparatus consisted of a Y-shaped acrylic maze with three equal-length arms (each with the dimensions 30 cm × 8 cm × 15 cm), wherein the angle between each pair of arms was 120°. Each tested mouse was placed at the end of any arm of the Y maze and left to explore freely for 8 min. Behavioral changes in the mouse were recorded by the camera system. The following indicators were recorded: the total number of entries (entry of all four feet into an arm was one entry) and the alternations (entry into each of the three arms consecutively as one alternation).

The Morris water maze test was performed according to a previous report.^26^ The system consisted of a water maze (150 cm in diameter, 50 cm in height, filled with white water at 22 °C, and surrounded with distal cues outside the maze), a camera system, and an X-eye animal behavior trajectory analysis system (Panlab SMART 2.5). The training phase was carried out continuously for three days, four times each day. During training, the mice were placed into the pool in four different starting points. After the mouse found the platform, it was allowed to stand on the platform for 10 s. If the mouse failed to find the platform within 60 s, it was gently dragged from the water onto the platform and allowed to stay for 10 s before proceeding to the next training. The speed, latency, distance to reach the platform and swim path of each trial were recorded by the video tracking system.

### Statistical analysis

All experiments were performed independently at least three times. The data are presented as the mean ± standard error of the mean (SEM). T-tests with

Welch’s correction or one-way ANOVA with the Sidak correction were used for parametric data analysis. Mann–Whitney tests or Kruskal–Wallis tests with Dunn’s correction were used for nonparametric data analysis. The log-rank test was used for survival curve analysis. *P* values of less than 0.05 were considered statistically significant. GraphPad Prism software was used for the calculations.

## Results

### CD146 is upregulated on BBBECs by inflammatory factors during eCM development

**Fig. 1:**
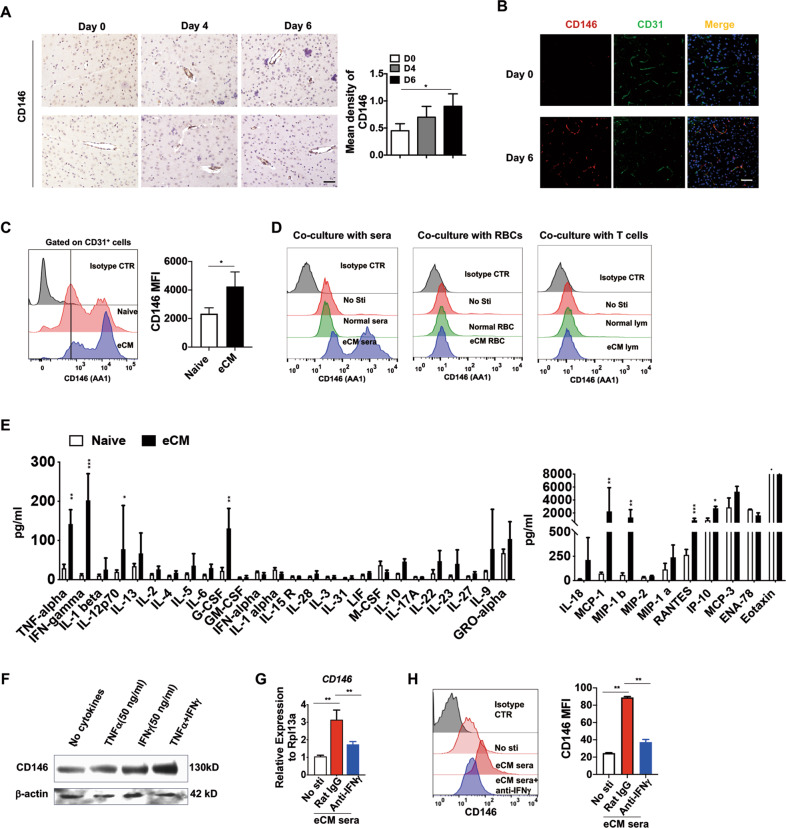
CD146 is upregulated on BBBECs during eCM development. **a** Immunohistochemical staining of CD146 (AA4) during eCM development. Scale bar, 50 µm. Right panel, the mean intensity of CD146 was calculated with ImageJ software. **b** Immunofluorescence analysis of CD146 (red) and CD31 (green) in paraffin-embedded brain sections from healthy (D0) and eCM mice (D6). Nuclei were counterstained with DAPI (blue). Scale bar, 50 µm. **c** FACS analysis of CD146 expression on BBBECs isolated from healthy and eCM mice (D6). Right panel, the mean fluorescence intensity (MFI) of CD146. **d** FACS analysis of CD146 expression in bEnd.3 cells cocultured with sera, RBCs, or T cells from healthy mice or eCM mice (D6). **e** ProcartaPlex immunoassays of 36 kinds of soluble factors in the sera of naive and eCM mice (D6) (*n* = 4). **f** Western blot analysis of CD146 protein expression in BBBECs after cytokine stimulation for 36 h. β-actin served as a loading control. CD146 mRNA (**g**) and protein (**h**) expression in BBBECs treated as indicated. Right panel, the mean fluorescence intensity (MFI) of CD146. *p < 0.05, **p < 0.01, ***p < 0.001. The data are representative of at least three independent experiments (**a**, **b**, **c**, **d**, **f**, **g**, and **h**)

To evaluate the essential role of CD146 in the development of eCM, we first assessed CD146 expression on BBBECs during the development of eCM. As shown in Fig. 1a, CD146 expression was reduced in the brains of naive mice and was upregulated on the fourth day after pRBC injection; expression remained high in eCM mice. In addition, CD146 expression in the brain was mainly localized on BBBECs. This result was confirmed via immunofluorescence. As shown in Fig. 1b, we found that eCM mice had higher expression of CD146 on CD31^+^ BBBECs than naive mice. Using flow cytometry, we further confirmed this finding by measuring CD146 expression in BBBECs that were isolated from naive mice and mice with eCM (Fig. 1c). These results suggest that the expression of CD146 is inducible under eCM conditions. To identify which factors induce CD146 expression on BBBECs, we cocultured the mouse BBBEC cell line bEnd.3 with sera, RBCs, and T cells isolated from infected mice and healthy control mice and then measured CD146 expression on BBBECs using flow cytometry. After 24 h of coculture, we found that only the sera from eCM mice induced CD146 overexpression (Fig. 1d), suggesting that inflammatory factors in infected sera promote CD146 expression.

To determine the exact factors involved in this process, we performed proinflammatory factor profile analysis using healthy sera and infected sera. We found that among the 36 tested factors, interferon γ (IFNγ), TNFα, interleukin-12 (IL-12), granulocyte colony-stimulating factor (G-CSF), interleukin-18 (IL-18), monocyte chemoattractant protein 1 (MCP-1), macrophage inflammatory protein 1 (MIP-1β), regulated upon activation normal T cell expressed and secreted factor (RANTES), and interferon-inducible protein-10 (IP-10) were significantly increased by five to ten fold during eCM development (Fig. 1e), suggesting that some of these factors contribute to the overexpression of CD146 on BBBECs. A previous study showed that inflammatory factors such as TNFα induce CD146 expression.^27^ We hypothesized that high levels of inflammatory factors in eCM upregulate endothelial CD146 expression. To confirm this hypothesis, we cultured BBBECs in vitro in the presence of IFNγ and TNFα and measured CD146 expression. As shown in Fig. 1f, CD146 expression was induced in response to both factors and reached a higher level when the stimuli were used in combination. This finding was confirmed by using anti-IFNγ antibodies. As shown in Fig. 1g, h, eCM sera upregulated the expression of CD146, whereas antibodies against IFNγ downregulated CD146 expression to some extent. These data suggest that the upregulation of CD146 on BBBECs was induced by inflammatory factors and may play an important role in the pathogenesis of CM.

### Increased numbers of CD146+ inflammatory cells during eCM development

**Fig. 2:**
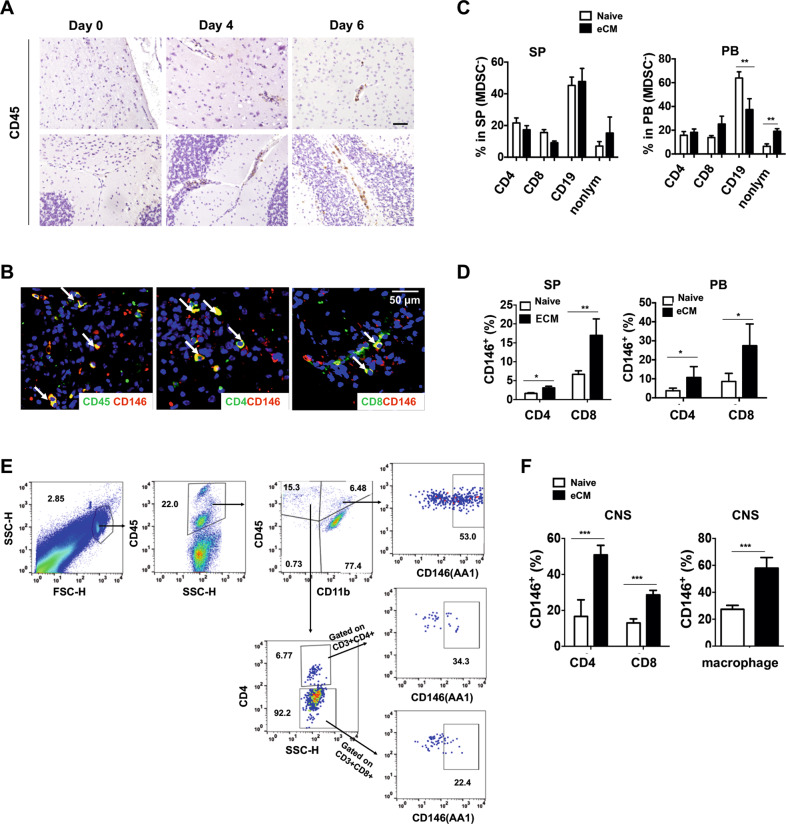
CD146^+^ T cells and macrophages are increased during eCM development. **a** Immunohistochemical staining of CD45 during eCM development. Scale bar, 50 µm. **b** Immunofluorescent staining of CD146 and CD45, as well as CD4 or CD8, in the brains of eCM mice. **c** The percentages of CD4^ +^ , CD8^+^ , and CD19^+^ cells in the MDSC (CD11b^−^Gr-1^−^) populations isolated from the spleen (SP) and peripheral blood (PB) of healthy or eCM mice (*n* = 10). **d** The percentages of CD146^+^ CD4 and CD8 T cells in the SP and PB of healthy and eCM mice (n = 10). **e** Representative flow cytometric analysis of brain infiltrated leukocytes. **f** The percentages of CD146^+^ CD4 and CD8 T cells, as well as CD146^+^ macrophages, in the brains of healthy and eCM mice (*n* = 5). *p < 0.05, **p < 0.01, ***p < 0.001. The sata are representative of at least three independent experiments

The number of infiltrated leukocytes in the brain were increased during eCM development (Fig. 2a). Consistent with the findings of many previous reports, we found that CD146 was expressed on subtypes of inflammatory cells (Fig. 2b), such as T cells and macrophages. To more fully understand the essential roles of CD146 in eCM development, we also isolated inflammatory cells and measured CD146 expression. As shown in Fig. 2c, compared with those of healthy controls, the percentages of T cells and B cells in eCM mice were not significantly changed. However, both CD146^+^ CD4^+^ T cells and CD146^+^ CD8^+^ T cells from eCM mice were markedly increased in the peripheral blood (PB) and spleen (Fig. 2d), while the percentages of CD146^+^ non-T cells were markedly reduced (data not shown). In addition, CD146^+^ T cells and CD146^+^ macrophages were increased in the CNS of eCM mice (Fig. 2e, f). These data suggest that both CD146^+^ T cells and CD146^+ ^macrophages may be involved in the pathogenesis of eCM.

### Endothelial CD146 but not lymphocytic or macrophagic CD146 promotes eCM development

**Fig. 3:**
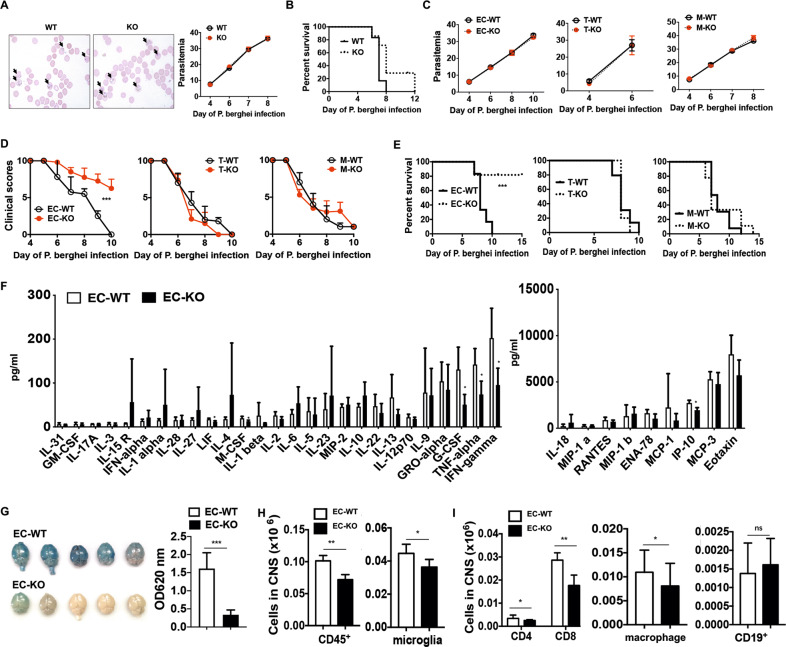
CD146 on ECs but not T cells or macrophages promotes eCM development. **a** Giemsa-stained pRBCs from CD146-WT and -KO eCM mice were observed under a 100-X oil-immersion lens (day 8 postinfection). The black arrow indicates the pRBC. Right panel, The percentages of parasitemia from day 4 to day 8 postinfection in WT and KO eCM mice (*n* = 6). **b** Survival analysis of CD146-WT and -KO eCM mice (*n* = 6). **c** The percentages of parasitemia in WT, EC-KO, T-KO, and M-KO eCM mice (*n* = 6). **d** Clinical scores of WT, EC-KO, T-KO, and M-KO eCM mice (*n* = 6–10). **e** Survival analysis of CD146 WT, EC-KO, T-KO, and M-KO eCM mice (*n* = 6–10). **f** Proinflammatory cytokine analysis of EC-WT and EC-KO eCM mice (*n* = 4). **g** Representative Evans blue-stained brains from EC-WT and EC-KO eCM mice at day 6 postinfection with Pb. ANKA. Right panel, OD620 values of Evans blue analysis of the brains of EC-WT and EC-KO eCM mice (*n* = 4). **h** CNS-infiltrated leukocyte analysis of EC-WT and EC-KO eCM mice (day 7 postinfection) (*n* = 6). *p < 0.05, **p < 0.01, ***p < 0.001. The data are representative of at least three independent experiments (**a**, **b**, **c**, **d**, **e**, **g**, **h**)

As mentioned previously, CD146 was upregulated on BBBECs, as well as on T cells and macrophages, during eCM development. To more fully elucidate the effects of CD146 on CM development, we first established an eCM model using CD146-knockout (KO) mice and their wild-type (WT) littermates. Surprisingly, we did not observe any significant difference in the clinical symptoms or survival between WT and KO mice (Fig. 3a, b). To assess the exact role of CD146^+^ cells in eCM development, we generated three kinds of CD146 conditional-knockout mice (see methods) that lacked CD146 on endothelial cells (EC-KO mice), T cells (T-KO mice), or macrophages (M-KO mice). The genotypes were confirmed by PCR analysis. All three genotypes cell-specific CD146 mRNA levels that were below the detection limit. We confirmed the CD146 protein knockout in the different mouse genotypes using flow cytometry (Supplementary Fig. 1). We established eCM models using these mice and their control littermates and evaluated their eCM symptoms. We found no significant difference in parasitemia among these groups (Fig. 3c). However, compared with that of WT mice, the clinical severity was alleviated in CD146 EC-KO mice but not T-KO mice or M-KO mice (Fig. 3d). Moreover, CD146 EC-KO mice had much lower mortality rates (20%) than the other mice, while T-KO mice and M-KO mice had 100% mortality rates (Fig. 3e). Consistent with these findings, CD146 EC-KO eCM mice had reduced levels of inflammatory factors, including TNFα, IFNγ, leukemia inhibitory factor (LIF), macrophage colony-stimulating factor (M-CSF), G-CSF, and IP-10 (Fig. 3f). In addition, CD146 EC-KO eCM mice had reduced CNS permeability (Fig. 3g). Moreover, the number of infiltrated inflammatory cells and resident microglial cells were reduced in CD146 EC-KO eCM mice (Fig. 3h). Among the infiltrated leukocytes, macrophages and lymphocytes, especially CD4^+ ^T cells and CD8^+^ T cells, were significantly reduced in CD146 EC-KO eCM mice, while CD19^+^ B cells were less profoundly affected than other cell types (Fig. 3i). These results showed that CD146 expressed on BBBECs but not T cells or macrophages was essential for the development of eCM.

### Endothelial CD146 is required for pRBC and inflammatory T cell adhesion to BBBECs

CD146 knockdown (CD146-KD) blocked the inflammatory factor-induced activation of BBBEC signaling pathways, such as NF-κB, p38, and Erk1/2 (Supplementary Fig. 2A). When cocultured with PbA-parasitized red blood cells (pRBCs), many chemokines and cytokines were increased in BBBECs. Knockdown of CD146 blocked the expression of these factors, whereas the overexpression of CD146 (CD146-OE) promoted their expression (Supplementary Fig. 2B). These data suggest that CD146 is important for BBBEC activation in eCM development.

**Fig. 4:**
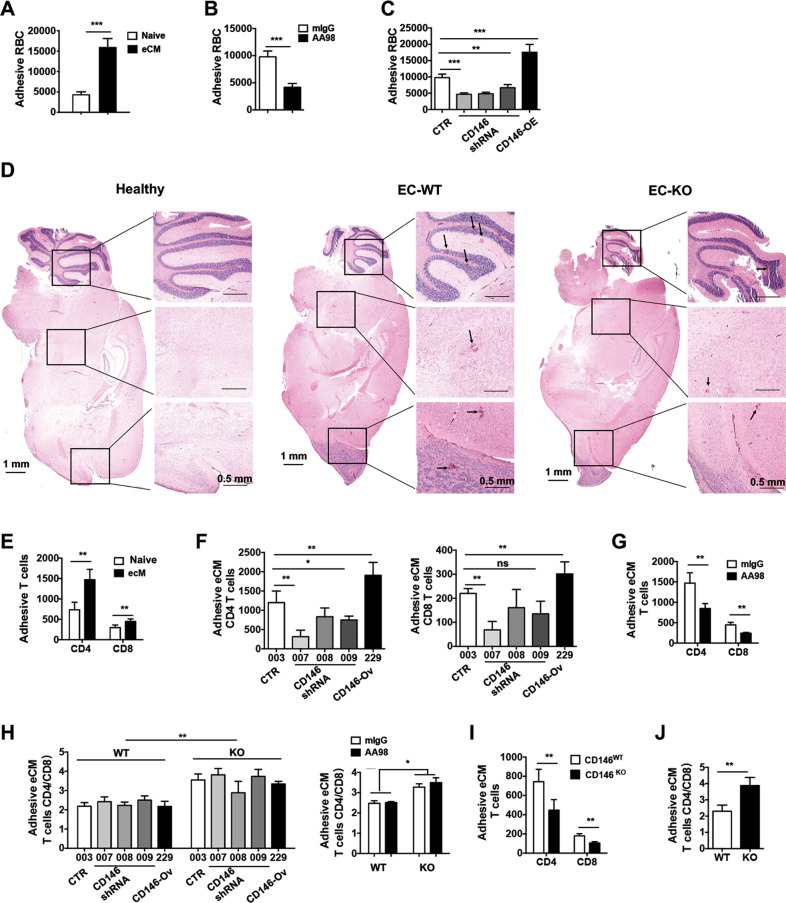
Endothelial CD146 is required for pRBC and inflammatory T cell adhesion to BBBECs. **a** Adhesive RBCs from eCM mice and healthy mice (*n* = 3). **b** Adhesive RBCs after CD146-specific antibody treatment (*n* = 3). **c** RBC adhesion to BBBECs with CD146 knockdown or overexpression (*n* = 3). **d** The sequestration of pRBCs in the brains of healthy and WT or EC-KO eCM mice. Black arrows indicate pRBCs. **e** Adhesive T cells from healthy and eCM mice (*n* = 3). **f** T cell adhesion to BBBECs with CD146 knockdown or overexpression (*n* = 3). **g** Adhesive T cells after CD146-specific antibody treatment (*n* = 3). **h** The ratios of CD4 and CD8 cells from CD146-WT and CD146-KO eCM mice that adhered to BBBECs with CD146 knockdown or overexpression (left) or after CD146-specific antibody treatment (right) (*n* = 3). **I** Adhesive T cells from CD146-WT and -KO eCM mice (*n* = 3). **j** The ratios of CD4 and CD8 cells from CD146-WT and CD146-KO eCM mice (*n* = 3). ns not significant, *p* > 0.05, *p < 0.05, **p < 0.01, ***p < 0.001. The data are representative of at least three independent experiments

It has been reported that pRBC and inflammatory T cell adhesion to BBBECs represents an essential characteristic of eCM development.^14^ To evaluate the important role of CD146 in adhesion during eCM development, we first measured the ability of pRBCs to adhere to BBBECs in the presence or absence of CD146 in vitro. The in vitro adhesion assays showed that pRBCs were more susceptible to BBBEC adhesion than healthy RBCs (Fig. 4a). The adhesion of pRBCs was blocked by the anti-CD146 functional antibody AA98 (Fig. 4b). To explore the essential role of CD146 on pRBC adhesion, we performed adhesion assays using bEnd.3 cells with either CD146 knockdown or overexpression. These cell lines were established as described in our methods, and CD146 expression levels were measured by flow cytometry (Supplementary Fig. 2C). As shown in Fig. 4c, more pRBCs adhered to CD146-overexpressing BBBECs, while fewer pRBCs adhered to CD146-knockdown BBBECs. This result was also confirmed in CD146 EC-KO eCM mice, which had fewer adherent pRBCs in CNS blood vessels than WT mice (Fig. 4d). Collectively, these data suggest that BBBEC CD146 is essential for pRBC adhesion during eCM development.

Many previous reports have shown that T cells, especially CD8^+^ T cells, promote eCM development by adhering to BBBECs.^28^ Hence, we next evaluated the important role of BBBEC CD146 in the adhesion of inflammatory T cells. As shown in Fig. 4e, compared with those from healthy mice, T cells isolated from eCM mice showed a strong capacity to adhere to BBBECs, which was consistent with the findings of previous reports. Importantly, the adhesion capacities of both CD4^+^ and CD8^+^ T cells were enhanced when BBBECs overexpressed CD146. However, BBBEC-specific knockdown of CD146 inhibited T cell adhesion (Fig. 4f). Blocking CD146 with the antibody AA98 also reduced T cell adhesion (Fig. 4g). These data suggest that CD146 on BBBECs promotes the adhesion of inflammatory T cells. In addition, this CD146-mediated promotion showed a similar effect on the adhesion of CD4^ +^ T cells and CD8^+^ T cells, as the ratio of CD4/CD8 was similar regardless of the absence or presence of CD146 on BBBECs. This finding was confirmed by treatment with the AA98 antibody (Fig. 4h). Interestingly, CD146 on T cells also promoted T cell adhesion to BBBECs, as T cells isolated from CD146-KO eCM mice showed reduced adhesion to BBBECs (Fig. 4i). Moreover, the effect of T cell CD146 was greater in CD8^+^ T cells than in CD4^ +^ T cells, as the ratio of adherent CD4/CD8 among CD146-KO T cells was increased (Fig. 4h, j). These data suggest that endothelial CD146-mediated promotion of inflammatory T cell adhesion was independent of CD146 expression on T cells, while CD146^+^ T cell adhesion to BBBECs at least partially depended on CD146 expression, especially on CD8^+^ T cells. Taken together, these results suggest that endothelial CD146 pRBC and inflammatory T cell adhesion to BBBECs during eCM development.

### The CD146/Galectin-9 axis promotes pRBC and inflammatory T cell adhesion to BBBECs

**Fig. 5:**
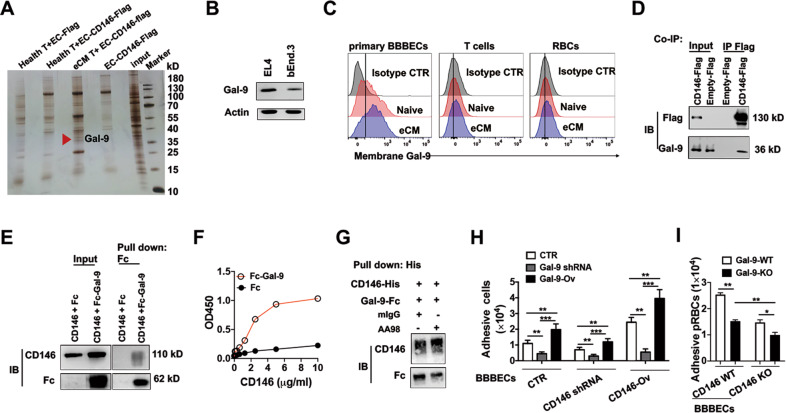
The CD146/Galectin-9 axis promotes pRBC and lymphocyte adhesion to BBBECs. **a** Silver staining of proteins from immunoprecipitation samples (*n* = 3). **b** Western blot analysis of Gal-9 expression in the EL4 and bEnd.3 cell lines. Actin served as a loading control. **c** FACS analysis of membrane-bound Gal-9 on primary BBBECs, T cells, and RBCs from healthy and eCM mice. **d** Immunoprecipitation analysis of the interaction between CD146 and Gal-9 in bEnd.3 and T cells. **e** Pull-down analysis using sCD146 and Gal-9-FC proteins. **f** Fc or Fc-Gal-9 was added to wells coated with different concentrations of CD146, and ELISA was performed. **g** Pull-down analysis of CD146 and Gal-9 in the presence of the anti-CD146 antibody AA98 or control mIgG antibody. **h** Adhesion assay of T cells with knockdown or overexpression of Gal-9 and bEnd.3 cells with knockdown or overexpression of CD146 (*n* = 3). **i** Adhesion assay of pRBCs isolated from Gal-9-WT or Gal-9-KO eCM mice and primary BBBECs isolated from CD146-WT or CD146-KO mice. *p < 0.05, **p < 0.01, ***p < 0.001. The data are representative of at least three independent experiments

To determine whether CD146 directly mediates pRBC and T cell adhesion to BBBECs, we performed an in vitro coimmunoprecipitation (co-IP) tandem-mass spectrometry assay using BBBECs that were stably transfected with CD146-Flag plasmid and T cells isolated from healthy or eCM mice. The results showed that galectin-9, a β-galactoside-binding lectin, was a candidate CD146-interacting protein (Fig. 5a). Galectin-9 has been reported to be increased in malaria patients^29^, suggesting that Galectin-9 promotes CM development. We measured the expression of Galectin-9 on BBBECs and T cells. We found that Galectin-9 was expressed on both BBBEC and T cell lines and was more highly expressed in the T cell line than the BBBEC line (Fig. 5b). Moreover, primary BBBECs, T cells, and pRBCs from eCM mice showed higher levels of membrane-bound Galectin-9 expression than those from healthy mice (Fig. 5c). Thus, CD146 may promote eCM development at least partially by associating with Galectin-9.

To confirm the interaction of CD146 and Galectin-9, we performed Co-IP experiments using antibodies and measured Galectin-9 protein levels by Western blotting. As shown in Fig. 5d, CD146 interacted with galectin-9. Moreover, a pull-down assay showed a direct interaction between CD146 and Galectin-9 (Fig. 5e). This finding was confirmed by ELISA (Fig. 5f). In addition, treatment with the antibody AA98 partially blocked this interaction in a pull-down assay (Fig. 5g). These data suggest that the CD146/Galectin-9 interaction contributes to the adhesion of BBBECs and T cells. To confirm this finding, we established galectin-9-knockdown and galectin-9-overexpression T cell lines using shRNAs and overexpression plasmids, respectively. The protein level of Galectin-9 was measured via both flow cytometry and Western blotting (Supplementary Fig. 3A–C). An in vitro adhesion assay showed that Galectin-9 knockdown significantly reduced T cell adhesion to BBBECs, while Galectin-9 overexpression increased T cell adhesion. In the absence of endothelial CD146, the effect of Galectin-9 was partially counteracted. CD146 overexpression partially ameliorated the adverse effect of the lack of Galectin-9 (Fig. 5h). This finding was confirmed by using primary Galectin-9-knockout (Gal-9-KO) pRBCs. As shown in Fig. 5i, compared with Gal-9 WT pRBCs, Gal-9-KO pRBCs showed a lower ability to adhere to primary BBBECs. In addition, CD146 knockout in BBBECs further inhibited the adhesion of Gal-9-KO pRBCs. Taken together, these data suggest that T cell adhesion to BBBECs at least partially depends on the interaction of CD146 and Galectin-9.

### Inhibition of CD146 via the CD146-specific functional antibody AA98 suppresses eCM development and prevents cognitive impairment in artemether-treated eCM mice

**Fig. 6:**
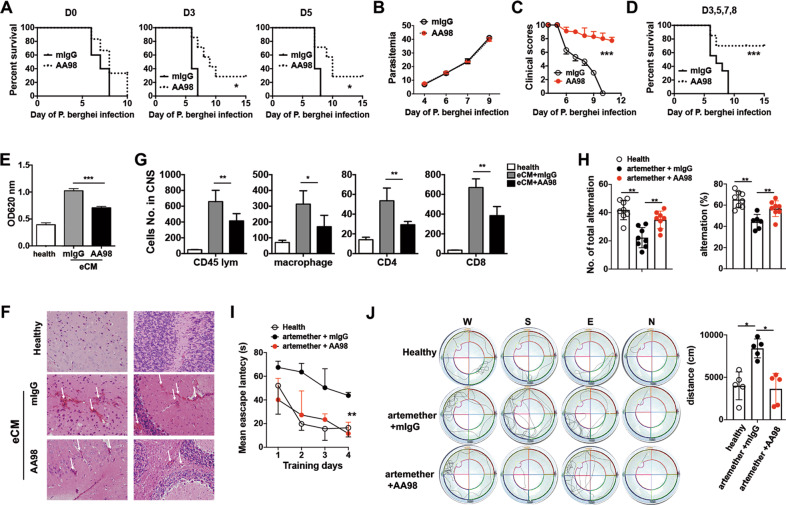
Targeting CD146 with the AA98 antibody suppresses eCM development and prevents cognitive impairment in artemether-treated eCM mice. **a** Survival analysis of eCM mice after a single treatment with the AA98 antibody on day 0, day 3, or day 5. **b** The percentages of parasitemia in eCM mice treated with antibodies (*n* = 6). **c** The clinical scores of eCM mice treated with antibodies (*n* = 10). **d** Survival analysis of eCM mice after AA98 antibody treatment on days 3, 5, 7 and 8 (*n* = 10). **e** The OD620 values of Evans blue in the brains of healthy mice treated with PBS and eCM mice treated with mIgG or AA98 antibodies (day 8) (*n* = 4). **f** The sequestration of pRBCs in the brains of healthy mice treated with PBS and eCM mice treated with mIgG or AA98 antibodies (day 8) (*n* = 3). White arrows indicate aggregated pRBCs. **g** CNS-infiltrated leukocytes from healthy mice treated with PBS and eCM mice treated with mIgG or AA98 antibodies (day 9) (*n* = 6). **h** The total alternations and successful alternations (%) in the three groups of mice in the Y maze test. The mean escape latency (**i**) and total distance to reach the platform (**j**) in the three groups of mice in the Morris water maze test. Each symbol represents a mouse. *p < 0.05, **p < 0.01, ***p < 0.001. The data are representative of at least three independent experiments

Because endothelial CD146 plays an important role in the adhesion of pRBCs and inflammatory cells, we next examined the therapeutic effect of the anti-CD146 antibody AA98 in eCM. We treated eCM mice with a single injection of AA98 and control mice with a single injection of the mouse IgG (mIgG) isotype on day 0, day 3, or day 5 after PbA infection, prior to or upon the onset of eCM symptoms (day 5). The results showed that AA98 treatment did not affect parasitemia. A single injection on day 0 after PbA infection did not exhibit an obvious effect on the survival of eCM mice. However, a single injection on day 3 or day 5 after PbA infection significantly increased the survival of eCM mice (Fig. 6a). These data suggest that CD146 is a potential therapeutic target after the onset of eCM. To further test this hypothesis, we administered either AA98 or control mIgG antibodies to mice with established on days 3, 5, 7, and 8 after PbA infection. There was no significant difference in parasitemia between the two groups (Fig. 6b). However, AA98-treated mice exhibited an alleviation in the severity of clinical symptoms (Fig. 6c). In addition, the mortality in the AA98-treated group was markedly reduced compared with that in the mIgG-treated group (Fig. 6d). Consistent with these findings, BBB permeability was reduced in AA98-treated mice (Fig. 6e). Hematoxylin and eosin (HE) staining revealed reduced pRBC sequestration in brain blood vessels following AA98 treatment (Fig. 6f). Analysis of CNS-infiltrated leukocytes via flow cytometry confirmed that AA98 treatment blocked CNS entry of inflammatory cells, including macrophages, CD4^+^ T cells, and CD8^+^ T cells (Fig. 6g). In addition, treatment with AA98 did not disturb the peripheral immune response or the numbers of platelets or RBCs (Supplementary Fig. 4A, B). Collectively, these data suggest that targeting CD146 with AA98 exerts a therapeutic effect by drastically suppressing the accumulation of pRBCs and inflammatory cells in the CNS, thereby ameliorating eCM symptoms.

To examine whether AA98 treatment protected against the impairment of cognitive functions in antiparasitic agent-treated eCM mice, we performed the Y maze and Morris water maze tests. eCM mice were treated with a combination of AA98 and artemether as described in the Methods section. Parasitemia was measured every other day after treatment (Supplementary Fig. 4C). In the Y maze test, compared with healthy mice, eCM mice treated with artemether and mIgG completed fewer total alternations and exhibited less successful alternations, whereas eCM mice treated with artemether and AA98 had much better alternation results (Fig. 6H). These data suggest that AA98 combined with the antiparasitic agent improved the learning and memory abilities of eCM mice. This finding was confirmed by using the Morris water maze test. As shown in Fig. 6I, as the training days increased, the mean escape latency shortened. The eCM mice treated with artemether and AA98 had shorter escape latencies than mice treated with artemether and mIgG. Moreover, AA98-treated mice had shorter total distances to reach the platform than mice in the other group (Fig. 6J). Overall, these data indicate that combining AA98 with the antiparasitic agent artemether prevents the cognitive impairment observed in artemether-treated eCM mice.

## Discussion

In the present study, we provide detailed evidence to support the hypothesis that BBB endothelial CD146 is a novel target for CM therapy. The expression of CD146 on BBBECs was induced under inflammatory conditions during the development of eCM. CD146 was actively involved in the sequestration of pRBCs and inflammatory lymphocytes by associating with galectin-9. Deletion of CD146 or antibody targeting of CD146 attenuated BBB disruption and CNS inflammation, thereby alleviating eCM symptoms and prolonging survival. Importantly, early targeting of CD146 also prevented lethal progression after the onset of eCM. Taken together, our findings indicate that CD146 is a promising candidate target for CM therapy.

A growing number of studies have shown that the disruption of BBB integrity is critical for CNS dysfunction and death in CM.^12^ However, the precise mechanisms by which the disruption of BBB integrity occurs during CM remain unclear. It has been proposed that several events are associated with BBB disruption, such as the sequestration of pRBCs by brain ECs,^14^ heightened intracerebral proinflammatory responses^13^, intravascular coagulation in the brain,^30^ and the dysregulation of vascular ECs.^31^ The constant and dynamic interplay among these events promotes BBB dysfunction, leading to neurological alterations and death. These infected cells express *P. falciparum* erythrocyte membrane protein-1 (PfEMP1), which mediates the binding of pRBCs to ECs by engaging various receptors on ECs, such as vascular cell-adhesion molecule-1 (VCAM-1), intercellular-adhesion molecule 1 (ICAM-1), CD36, and cytokine-activated endothelial protein C receptor (EPCR).^32,33^ The engagement of receptors induces remodeling of the endothelial cytoskeleton to modulate BBB permeability. In addition, EC activation induces the expression of various adhesion molecules that promote the intracerebral recruitment of leukocytes, including macrophages and CD8^+^ T cells.^34^ Infiltrated leukocytes mediate BBB disruption by inducing EC apoptosis through granzyme B and perforin-mediated cytotoxicity. In the present study, we show that CD146 is involved in BBB dysfunction during eCM development. The upregulation of CD146 on BBBECs mediates the binding of pRBCs to BBBECs, facilitating the sequestration of pRBCs. Moreover, this binding is not dependent on plasmodium-specific protein expression. In CM patients, the plasmodium-specific protein PfEMP1 is encoded by a family of approximately 60 var genes. Various var genes generate different forms of PfEMP1, which aid in parasite escape from host immune defense. Studies have shown that these receptors on ECs bind different versions of PfEMP1,^35–37^ leading to the limited effect of monotherapy. Therefore, a treatment that is independent of PfEMP1 expression is expected to be superior to a treatment that is dependent on PfEMP1 expression. In addition, CD146 promotes the intracerebral accumulation of multiple leukocytic populations, including macrophages and T cells, exacerbating local inflammation and thereby potentiating the disruption of BBB integrity. Thus, targeting CD146 may have a dual role in preventing BBB dysfunction.

Our present study also provides evidence that the effect of CD146 on adhesion partially depends on Galectin-9. Lacking a typical transmembrane domain, a soluble form of galectin-9 is often secreted extracellularly. The membrane-bound form has also been observed in T cells.^38^ It has been reported that the binding of Galectin-9 to glycoproteins on immune cells exerts a variety of pathological effects, such as cell adhesion and death.^39^ Increased levels of plasma Galectin-9 have been observed in patients with several types of malaria,^29^ but the biological roles of Galectin-9 in malaria have remained unclear. In the present study, we showed that the binding of CD146 to Galectin-9 facilitates the adhesion of BBBECs and T cells. Galectin-9 has several receptors, such as Tim-3. The Gal-9–Tim-3 interaction may also play a role in T cell adhesion to ECs.^40^ Nevertheless, the interaction of CD146 and Gal-9 is the major axis in the adhesion of pRBCs and T cells, and blocking this interaction inhibits a large amount of cell adhesion. It has been reported that domain 5 of the CD146 protein contains poly-N-acetyllactosamine sites, namely, N418, N449, and N544, and that the glycosylation of CD146 promotes the binding of CD146 to Galectin-3 and Galectin-1.^41–44^ We suggest that the glycosylation of CD146 domain 5 also contributes to the binding of CD146 to Galectin-9, which is supported by the following evidence: A functional antibody against CD146, AA98, has been reported to act at domains 4–5 of CD146.^45^ In our present study, treatment with AA98 significantly reduced the interaction of CD146 with Galectin-9. Therefore, AA98 may be a promising candidate for CM therapy. However, whether the interaction between CD146 and Galectin-9 directly depends on the glycan structure and how AA98 interferes with this interaction require further investigation.

CD146 has been reported to be involved in many inflammatory diseases, including MS,^18,19,46^ atherosclerosis,^20^ rheumatoid arthritis,^47,48^ Crohn’s disease,^49^ and vasculitis.^50^ Many studies, including ours, have shown that CD146 is expressed on both endothelial cells and proinflammatory leukocytes under various inflammatory conditions.^18,19^ As a marker of angiogenesis, CD146 actively participates in the activation, cytoskeletal remodeling, and migration of endothelial cells. In proinflammatory cells, such as activated T cells^19^ and macrophages,^20^ CD146 is thought to be associated with the activation of proinflammatory cells. In the present study, during eCM development, we observed the expression of CD146 on BBBECs and proinflammatory cells, including T cells and macrophages. However, deletion of CD146 on T cells or macrophages did not prevent the progression of eCM, suggesting that CD146 + T cells and macrophages are not the primary factors contributing to BBB dysfunction. CD146 + T cells have been reported to be a population of proinflammatory lymphocytes that secrete multiple cytokines that mediate the development of inflammation,^19^ such as in MS. Blocking the entry of CD146^+^ T cells into the CNS is a potential MS therapeutic strategy.^46,51^ However, in the eCM model in our present study, deletion of CD146^+^ T cells did not show any beneficial effect on the prevention of eCM. These data suggest that CD146 mediates T cell activation or function in immune responses to eCM. Macrophages play an important role in the clearance of parasites and microorganisms. CD146 is upregulated on macrophages and correlates with macrophage activation in lung infections.^52^ In our eCM model, deletion of CD146 on macrophages did not prevent the onset of disease, possibly due to the loss of macrophage ability to clear parasites, which needs further study. Interestingly, although there was no effect on parasitemia, deletion of BBBEC CD146 significantly alleviated eCM symptoms, indicating an important effect of endothelial CD146 on BBB integrity. In addition, targeting CD146 with the CD146-specific antibody AA98 after the onset of eCM prolonged survival. We also observed that combining AA98 and antiparasitic drugs prevented long-lasting neurological sequelae and memory impairments. In our previous study, we showed that AA98 did not significantly disturb the host’s protective immune response.^18^ Therefore, we suggest that targeting endothelial CD146 with AA98 represents a promising approach in combination with other antiparasitic drugs for CM treatment.

In summary, our findings reveal an important role of endothelial CD146 in BBB disruption during eCM development and suggest that targeting CD146 is a promising approach for CM therapy in combination with other antiparasitic drugs.

## Supplementary information

Supplemental Figure
