# Supplementary material for: Targeting the CD146/Galectin-9 axis protects the integrity of the blood–brain barrier in experimental cerebral malaria
Source: Cell Mol Immunol. 2020 Nov 17;18(10):2443–54. doi: 10.1038/s41423-020-00582-8 (PMC8484550; doi:10.1038/s41423-020-00582-8)
Supplement: Supplementary file 1 — Supplemental Figure [file 41423_2020_582_MOESM1_ESM.docx]

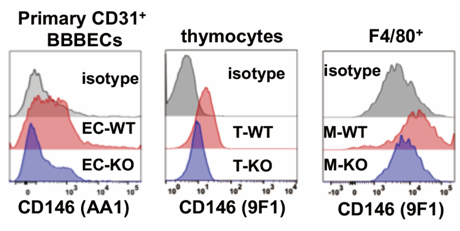


**Supplemental Figure 1. FACS analysis of CD146 protein levels in primary BBBECs, thymocytes, and F4/80^+^ cells from different conditional CD146 knockout mice.**


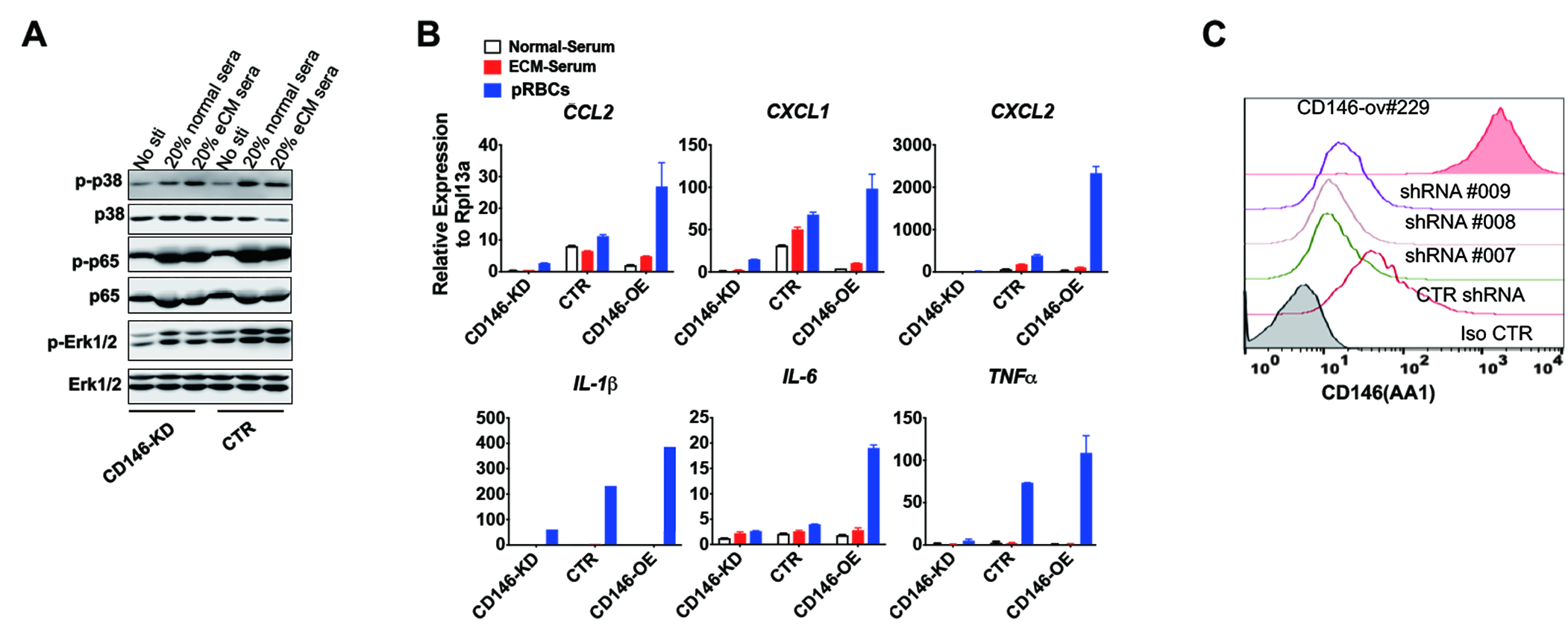


**Supplemental Figure 2. Endothelial CD146 is required for the adhesion of pRBCs and inflammatory T cells onto BBBECs. (A)** Wester blotting analysis of phosphorylation levels of p38, p65 and Erk1/2 in bEnd.3 cells treated with or without sera from eCM mice. Total p38, p65 and Erk1/2 were served as control. **(B)** Relative mRNA levels of chemokines and cytokines of BBBECs treated with sera or iRBCs for 24 h. **(C)** FACS analysis of CD146 protein levels in bEnd.3 cells.

**
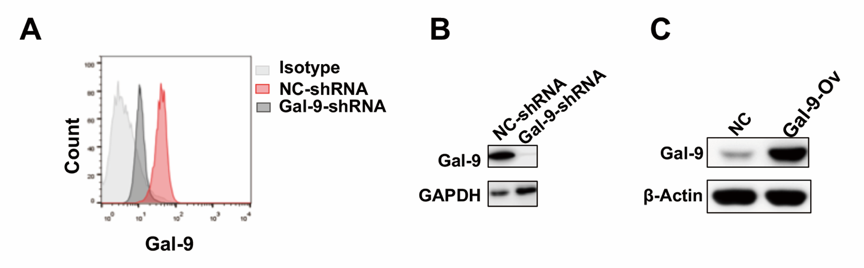
**

**Supplemental Figure 3. The CD146/Galectin-9 axis promotes the adhesion of pRBCs and lymphocytes onto BBBECs. (A–C)** FACS (**A**) and Western-blot (**B, C**) analysis of Gal-9 expression in EL4 cells under the indicated treatments.


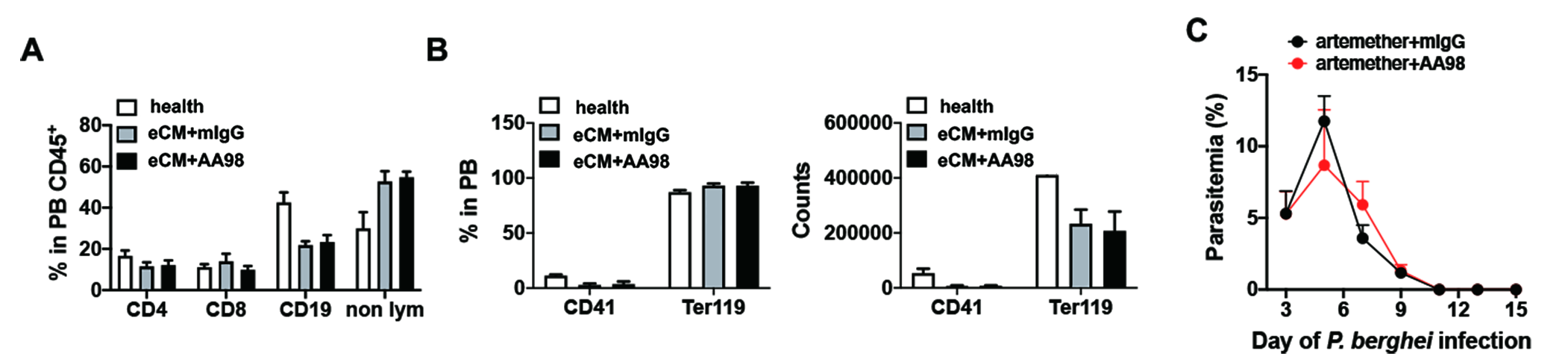


**Supplemental Figure 4. AA98 treatment does not disturb the peripheral immune response or the numbers of platelets or RBCs**. (**A**) Percentages of CD4, CD8, CD19, and non-T and non-B cells in the PB. (**B**) Percentages and numbers of CD41+ and Ter119+ cells in the PB. **(C)** The parasitemia of eCM mice treated with anti-parasite agent and antibody (n=8).
